# Second malignant neoplasms after treatment of 1487 children and adolescents with acute lymphoblastic leukemia—A population‐based analysis of the Austrian ALL‐BFM Study Group

**DOI:** 10.1002/jha2.488

**Published:** 2022-06-12

**Authors:** Fiona Poyer, Karin Dieckmann, Michael Dworzak, Melanie Tamesberger, Oskar Haas, Neil Jones, Karin Nebral, Stefan Köhrer, Reinhard Moser, Gabriele Kropshofer, Christina Peters, Christian Urban, Georg Mann, Ulrike Pötschger, Andishe Attarbaschi, the Austrian Berlin‐Frankfurt‐Münster (BFM) Study Group

**Affiliations:** ^1^ Department of Pediatric Hematology and Oncology, St. Anna Children's Hospital Medical University of Vienna Vienna Austria; ^2^ Department of Radiotherapy Medical University of Vienna Vienna Austria; ^3^ St. Anna Children's Cancer Research Institute (CCRI) Vienna Austria; ^4^ Department of Pediatrics and Adolescent Medicine Kepler University Hospital Linz Linz Austria; ^5^ Labdia Diagnostics Vienna Austria; ^6^ Department of Pediatrics and Adolescent Medicine University Clinics Salzburg Salzburg Austria; ^7^ Department of Pediatrics and Adolescent Medicine State Hospital Leoben Leoben Austria; ^8^ Division of Pediatric Hematology and Oncology and Stem Cell Transplantation, Department of Pediatrics and Adolescent Medicine Medical University of Innsbruck Innsbruck Austria; ^9^ Division of Pediatric Hematology and Oncology, Department of Pediatrics and Adolescent Medicine Medical University of Graz Graz Austria

**Keywords:** acute lymphoblastic leukemia, incidence, outcome, radiotherapy, second malignant neoplasms

## Abstract

Second malignant neoplasms (SMN) after primary childhood acute lymphoblastic leukemia (ALL) are rare. Among 1487 ALL patients diagnosed between 1981 and 2010 in Austria, the 10‐year cumulative incidence of an SMN was 1.1% ± 0.3%. There was no difference in the 10‐year incidence of SMNs with regard to diagnostic‐, response‐ and therapy‐related ALL characteristics except for a significantly higher incidence in patients with leukocytes ≥50.0 G/L at ALL diagnosis (2.1% ± 1.0% vs. 0% for 20.0–50.0 G/L, and 1.0% ± 0.3% for < 20.0 G/L; *p* = 0.033). Notably, there was no significant difference in the incidence of SMNs between patients with or without cranial radiotherapy (1.2% ± 0.5% vs. 0.8% ± 0.3%; *p* = 0.295). Future strategies must decrease the incidence of SMNs, as this event still leads to death in one‐third (7/19) of the patients.

## 1 INTRODUCTION

Acute lymphoblastic leukemia (ALL) is the most common malignancy in childhood and adolescence [1]. With 5‐year overall survival (OS) rates reaching 90%, the number of long‐term survivors has risen, and it becomes increasingly important to not only focus on leukemia‐free survival, but also on the quality of survival by evaluating the long‐term toxicity of pediatric ALL treatment [1, 2, 3]. Long‐term ALL survivors may suffer from chronic health conditions, ranging from organ dysfunctions to the development of secondary malignant neoplasms (SMN) [3, 4, 5]. SMNs cause considerable morbidity and, after relapse, are the main causes of death for ALL patients, making it imperative to reduce risk factors for their development without compromising ALL treatment efficacy [6]. Causes of SMNs are not fully clear, but seem to be due to an interplay of germline genetic variants in cancer predisposition genes and type of treatment, including cumulative cytotoxic drug and radiotherapy (RT) dosages [7, 8]. Herein, we present data on incidence, type, risk factors and outcome of SMNs in a population‐based cohort of pediatric ALL patients treated according to Berlin–Frankfurt–Münster (BFM)‐based protocols in Austria [9, 10, 11].

## 2 PATIENTS AND METHODS

Between January 1981 and December 2009, 1487 children and adolescents < 23‐years‐old with newly diagnosed ALL were enrolled in one of six multicenter trials in Austria (A): ALL‐BFM‐A 81 (*n* = 141), ALL‐A 84 (*n* = 127), ALL‐BFM‐A 86 (*n* = 142), ALL‐BFM‐A 90 (*n* = 256), ALL‐BFM‐A 95 (*n* = 230), and ALL‐BFM‐A 2000 (*n* = 591). Median follow‐up was 9.5 years (Q1‐Q3: 5.2‐13.1 years; Supporting information: Table S18). All patients were registered at the national study center in Vienna (St. Anna Children's Hospital and St. Anna Children's Cancer Research Institute), and events such as relapse, death or SMNs as well as dates of last‐follow‐up were either reported ad hoc by the respective treatment centers or during regular follow‐up queries/late effects screening performed by the national study center, and systematically recorded for the respective trials. Data collected on primary ALL disease included parameters with regard to demographics, response, treatment, and outcome. SMNs were defined as a non‐lymphoid malignancy or, in selected central nervous system (CNS) tumors, also as non‐malignant neoplasms (i.e., meningiomas). Notably, non‐melanoma skin cancers were not included in this analysis. In case of multiple SMNs, only the first SMN was used for primary analysis.

The aim of this study was to determine the incidence and characteristics of SMNs as a first event for all children treated with BFM‐based treatments for primary ALL in either of the 6 trials. Hence, as relapse was considered a competing risk, SMNs after ALL relapse were not considered as an event of interest. Data collected about the SMNs included clinical, histological, therapy, and outcome parameters. Details of the ALL‐treatment protocols, RT, and cumulative cytotoxic drugs of the respective ALL trials are included in the Supporting information (Tables S1‐S17) [9, 10, 11]. All patients were treated with informed consent of their legal guardian(s). Studies were conducted according to the Declaration of Helsinki, approved by the respective ethics committees and, since trial ALL‐BFM‐A 2000, registered at clinicaltrials.gov (NCT00430118).

## 3 STATISTICAL ANALYSIS

Overall survival of the patients with an SMN was defined as the time from diagnosis to death from any cause or date of last follow‐up. Event‐free survival (EFS) of the whole cohort and subsets of ALL patients were defined as the time from diagnosis to the first event (relapse, death, SMN) or date of last follow‐up. Survival rates were analyzed according to the Kaplan–Meier method and compared by the log‐rank test. In the calculation of the 10‐year cumulative incidence (CI) of SMNs, other failures such as relapse and death were treated as competing events. The CI of these competing events was also calculated. As allogeneic hematopoietic stem cell transplantation in first complete remission was not part of the ALL‐therapy protocols used, apart from a few defined indications in trial ALL‐BFM‐A 2000, transplantation was not considered a competing event. CI functions were constructed by the method of Kalbfleisch and Prentice and compared by the Grey's test. *P*‐values ≤0.05 were referred to as statistically significant.

## 4 RESULTS AND DISCUSSION

Nineteen of the 1487 patients (1.3%) developed an SMN as a first event, with all of them occurring after completion of primary ALL therapy (Table S19). One patient developed a further neoplasm (first SMN: primitive neuroectodermal tumor of the pelvis, subsequent SMN: acute myeloid leukemia), in four of the 1487 patients, an SMN developed after ALL relapse therapy (Supporting information: Table S18). Leukemia‐related initial characteristics, trial, chemo‐ and RT composition, early response during and after completion of ALL induction and consolidation therapy, and final risk group of the 19 patients with and 1468 patients without an SMN as a first event are shown in Tables 1A, 1B, 1C. The 10‐year CI of an SMN with death and relapse as competing events among the 1487 patients was 1.1% ± 0.3% (Table 2A, Figure S1). The 10‐year CI of first relapses, death as a first event and SMNs with deaths as the only competing event, are shown in Figures S2–S4. There was no statistically significant difference in the 10‐year CI of an SMN with regard to leukemia‐associated parameters such as the underlying trial, trial periods, gender, age, CNS status, genetics, immunophenotype, chemotherapy, cytomorphological response during and after induction therapy, minimal residual disease, and final risk group (Tables 2A, 2B, 2C). Only patients with higher leukocyte counts (≥50.0 G/L) had a significantly higher 10‐year CI of an SMN (Table 2A, *p* = 0.033). In addition, we run a model to assess the risk of SMNs, including relapse and death as competing events and the most relevant parameters such as the trial enrolled into, age at ALL diagnosis, leukocyte counts, and CRT to assess hazard ratios and confidence intervals, but did not find any of the parameters to be statistically relevant (Supporting information: Table S20).

**TABLE 1A:** Initial characteristics of ALL patients with and without an SMN as a first event

	**Number of patients with SMN**	**Number of patients without SMN**
**Number of patients**	19	1468
**Trial**		
ALL‐BFM‐A 81	1 (5%)	140 (10%)
ALL‐A 84	5 (26%)	122 (8%)
ALL‐BFM‐A 86	3 (16%)	139 (9%)
ALL‐BFM‐A 90	3 (16%)	253 (17%)
ALL‐BFM‐A 95	1 (5%)	229 (16%)
ALL‐BFM‐A 2000	6 (32%)	585 (40%)
Earlier era (81, 84, 86)	9 (47%)	401 (27%)
Later era (90, 95, 2000)	10 (53%)	1067 (73%)
Very early era (81, 84)	6 (32%)	262 (18%)
Later era (86, 90, 95, 2000)	13 (68%)	1206 (82%)
**Gender**		
male	9 (47%)	817 (56%)
female	10 (53%)	651 (44%)
**Age (years)**		
median	5.2	5.0
range	1.5–15.4	0.1–23.1
≥10 years	5 (26%)	331 (23%)
0–10 years	15 (74%)	1137 (77%)
**WBC count (G/L)**		
Median	14.0	10.4
Range	1.5–720.0	0.4–955.0
≥20.0	7 (37%)	521 (35%)
< 20.0	12 (63%)	947 (65%)
≥50.0	9 (47%)	295 (20%)
< 50.0	12 (63%)	1173 (80%)
**CNS disease**		
Negative	17 (89%)	1408 (96%)
Positive	1 (5%)	48 (3%)
Not available	1 (5%)	12 (1%)
**Immunophenotype**		
BCP‐ALL	16 (84%)	1223 (83%)
T‐ALL	3 (16%)	195 (13%)
Not available	0	50 (3%)
**Genetics**		
*ETV6::RUNX1*		
Positive	4 (21%)	260 (18%)
Negative	9 (47%)	946 (64%)
Not available	6 (32%)	262 (18%)
*TCF3::PBX1*		
Positive	0	37 (2%)
Negative	13 (68%)	1169 (80%)
Not available	6 (32%)	262 (18%)
*BCR::ABL1*		
Positive	0	25 (2%)
Negative	13 (68%)	1181 (80%)
Not available	6 (32%)	262 (18%)
*KMT2A*‐rearrangement		
Positive	1 (5%)	30 (2%)
Negative	12 (63%)	1176 (80%)
Not available	6 (32%)	262 (18%)
High‐hyperdiploidy		
Positive	2 (11%)	291 (20%)
Negative	10 (53%)	811 (55%)
Not available	7 (37%)	362 (25%)

Abbreviations: BCP, B‐cell precursor; CNS, central nervous system; SMN, secondary malignant neoplasm; WBC count, white blood cell count.

**TABLE 1B:** Early response and risk group of ALL patients with and without an SMN as a first event

	**Number of patients with SMN**	**Number of patients without SMN**
**Number of patients**	19	1468
**Prednisone response**		
Good	11 (58%)	1079 (74%)
Poor	2 (11%)	115 (8%)
Not available	6 (32%)	274 (19%)
**BM response on day 15**		
M1	5 (26%)	602 (41%)
M2	3 (16%)	313 (21%)
M3	1 (5%)	117 (8%)
not available	10 (53%)	436 (30%)
**Remission status on day 33**		
CR	13 (68%)	1161 (79%)
No CR	0	30 (2%)
Not available	6 (32%)	277 (19%)
**MRD group**		
Low‐risk	2 (11%)	154 (11%)
Intermediate‐risk	4 (21%)	358 (24%)
High‐risk	0	32 (2%)
Not available	13 (68%)	924 (63%)
**Final risk group**		
Standard‐risk	5 (26%)	516 (35%)
Intermediate‐risk	11 (58%)	748 (51%)
High‐risk	3 (16%)	185 (13%)
Not available	0	19 (1%)
Low‐risk	16 (84%)	1263 (86%)
High‐risk	3 (16%)	186 (13%)
Not available	0	19 (1%)
**Allogeneic HSCT**		
Yes	4 (21%)*	160 (11%)*
No	15 (79%)	1308 (89%)

Abbreviations: BM, bone marrow; CR, complete remission; HSCT, hematopoietic stem cell transplantation; MRD, minimal residual disease; SMN, secondary malignant neoplasm.

* All four HSCTs among the pts. with an SMN were performed in CR1, while the 160 HSCTs among the pts. without an SMN included HSCTs in CR1 as well as in ≥CR2.

**TABLE 1C:** Radio‐ and chemotherapy of ALL patients with and without an SMN as a first event

	**Number of patients with SMN**	**Number of patients without SMN**
**Number of patients**	19	1468
**Cyclophosphamide**		
≥3.000 mg/m^2^	18 (95%)	1366 (93%)
< 3.000 mg/m^2^	1 (5%)	81 (6%)
Not available	0	21 (1%)
**Cranial radiotherapy**		
Yes	14 (74%)	657 (45%)
No	5 (26%)	778 (53%)
Not available	0	33 (2%)
**VP‐16/VM‐26**		
Yes	2 (11%)	159 (11%)
No	17 (89%)	1288 (88%)
Not available	0	21 (1%)

Abbreviations: SMN, secondary malignant neoplasm; VM‐26, teniposide; VP‐16, etoposide.

Characteristics of the 19 patients with an SMN are summarized in Tables 1D and 1E. Six patients (32%) developed a hematologic SMN, nine (47%) a CNS tumor, and four (21%) suffered from “other” SMNs. All patients with hematologic SMNs originally suffered from B‐cell‐precursor ALL, as did eight of nine patients in the CNS tumor group and two of the four patients with “other” SMNs. The median time from ALL diagnosis to the diagnosis of an SMN was 3.5 years for hematologic, 10.2 years for CNS, and 9.6 years for “other” SMNs, respectively (see Table 1E).

**TABLE 1D:** Characteristics of the 19 primary ALL patients with a secondary malignant neoplasm as a first event

**Pt. Number**	**Study**	**Age at ALL (years)**	**Gender**	**Phenotype of ALL**	**Age at SMN (years)**	**Time to SMN (years)**	**Cranial radiotherapy**	**Type of SMN**	**Therapy of SMN** *	Outcome of SMN	Survival time from SMN (months)
1	ALL‐BFM‐A 81	10.2	m	C‐ALL	13.7	3.6	24 Gy	AML M1	1	dead (progression of SMN)	4
2	ALL‐A 84	3.1	m	C‐ALL	13.6	10.6	18 Gy	Thyroid carcinoma	2	alive	143
3	ALL‐A 84	10.4	f	C‐ALL	33.2	22.8	18 Gy	Meningioma	2	alive	2 (lost to FU)
4	ALL‐A 84	5.2	m	T‐ALL	19.4	14.2	18 Gy	Astrocytoma	1,2,3	dead (progression of SMN)	23
5	ALL‐A 84	2.8	f	C‐ALL	14.9	12.2	18 Gy	Meningioma	2	alive	108
6	ALL‐A 84	3.4	m	C‐ALL	13.6	10.2	18 Gy	Astrocytoma	2,3	dead (progression of SMN)	11
7	ALL‐BFM‐A 86	7.7	m	C‐ALL	12.0	4.5	12 Gy	AML M1	1,4	alive	105
8	ALL‐BFM‐A 86	3.5	f	C‐ALL	7.1	3.8	12 Gy	PNET, pelvis	1,2,3	dead (AML as 2. SMN)	21
9	ALL‐BFM‐A 86	8.5	f	T‐ALL	28.7	20.2	18 Gy	CCCA (Hep. C)	unknown	dead (progression of SMN)	9
10	ALL‐BFM‐A 90	2.6	f	C‐ALL	11.2	8.6	12 Gy	Glioblastoma multiforme	1,2,3	dead (progression of SMN)	14
11	ALL‐BFM‐A 90	1.5	f	C‐ALL	6.7	5.3	12 Gy	Ewing‘s sarcoma, occipital	1,2	alive	120
12	ALL‐BFM‐A 90	2.8	m	C‐ALL	9.6	6.9	12 Gy	PNET, brain	1,2,3	alive	87
13	ALL‐BFM‐A 95	3.3	m	C‐ALL	6.5	3.3	no	AML M4	1,4	alive	33
14	ALL‐BFM‐A 2000	11.7	f	C‐ALL	14.2	2.5	no	MDS ‐ RAEB ‐ T	1,4	alive	70
15	ALL‐BFM‐A 2000	15.4	m	T‐ALL	24.1	8.7	12 Gy	Osteosarcoma (right femur)	1,2	alive	lost to FU
16	ALL‐BFM‐A 2000	5.5	m	C‐ALL	9.2	3.6	no	MDS ‐ RAEB	1,4	alive	68
17	ALL‐BFM‐A 2000	11.2	f	C‐ALL	14.8	3.6	no	Astrocytoma	1,2,3	dead (progression of SMN)	9
18	ALL‐BFM‐A 2000	8.7	f	C‐ALL	11.6	3.0	no	CMML	1,4	alive	17
19	ALL‐BFM‐A 2000	4.0	f	pre‐B‐ALL	14.4	10.4	no	PNET, brain	1,2	alive	16

* Therapy of SMN: 1 = chemotherapy, 2 = operation, 3 = radiation, 4 = SCT = stem cell transplantation.

Abbreviations: AML, acute myeloid leukaemia; C‐ALL, common ALL; CCCA, cholangio‐cellular carcinoma; CMML, chronic myelomonocytic leukaemia; f, female; FU, follow‐up; Hep. C, hepatitis C; m, male; MDS, myelodysplastic syndrome; PNET, primitive neuroectodermal tumor; Pt, patient; RAEB, refractory anemia with excess blasts.

**TABLE 1E:** Characteristics of the three secondary malignant neoplasm subgroups

	**Hematologic SMN**	**Central nervous system tumors**	**“Other” SMN**
Number of patients	6	9	4
Male:female ratio	4:2	3:6	2:2
Median age at ALL (years)	8.1	3.4	6.1
Range (years)	3.3–11.7	1.5–11.2	3.1–15.4
BCP‐ALL	6	8	2
T‐ALL	0	1	2
Median time to SMN (years)	3.5	10.2	9.6
Range (years)	2.5–4.5	3.6–22.8	3.8–20.2
Median age at SMN (years)	11.8	14.4	18.9
Range (years)	6.5–14.2	6.7–33.2	7.1–18.7

Abbreviations: BCP‐ALL, B‐cell precursor ALL; SMN, secondary malignant neoplasms.

**TABLE 2A:** 10‐year CI of an SMN and competing events and event‐free survival according to the initial characteristics

		**Secondary malignancies**			**Competing events**			Event‐free survival	
**Parameters**	**Pts**.	**Events**	**10‐year CI**	***p*‐Value**	**Events**	**10‐year CI**	***p*‐Value**	10‐year EFS	p‐value
All patients	1487	19	1.1% ± 0.3%		328	24.0% ± 1.2%		75.0 ± 1.2%	
Trial									
ALL‐BFM‐A 81	141	1	0.7% ± 0.7%	0.202	59	42.8% ± 4.2%	<0.001	56.5 ± 4.2%	<0.001
ALL‐A 84	127	5	0.0% ± 0.0%		44	34.2% ± 4.2%		65.8 ± 4.2%	
ALL‐BFM‐A 86	142	3	1.4% ± 1.0%		33	22.6% ± 3.5%		76.0 ± 3.6%	
ALL‐BFM‐A 90	256	3	1.3% ± 0.7%		61	24.1% ± 2.7%		74.7 ± 2.7%	
ALL‐BFM‐A 95	230	1	0.4% ± 0.4%		48	21.1% ± 2.7%		78.5 ± 2.7%	
ALL‐BFM‐A 2000	591	6	2.2% ± 1.3%		83	18.3% ± 2.0%		79.5 ± 2.4%	
Earlier era (81, 84, 86)	410	9	0.7% ± 0.4%	0.983	136	33.1% ± 2.3%	<0.001	66.2 ± 2.4%	<0.001
Later era (90, 95, 2000)	1077	10	1.3% ± 0.4%		192	20.5% ± 1.4%		78.3 ± 1.4%	
Gender									
Male	826	9	1.0% ± 0.4%	0.544	185	24.6% ± 1.6%	0.82	74.4 ± 1.6%	0.939
Female	661	10	1.2% ± 0.5%		143	23.1% ± 1.7%		75.7 ± 1.8%	
Age (years)									
< 1	25	0	0.0% ± 0.0%	0.559	16	65.3% ± 9.7%	<0.001	34.7 ± 9.7%	<0.001
1–10	1126	14	0.9% ± 0.3%		213	20.6% ± 1.3%		78.5 ± 1.3%	
≥10	336	5	1.7% ± 0.9%		98	32.2% ± 2.8%		65.8 ± 2.8%	
WBC count (G/L)									
< 20.0	959	12	1.0% ± 0.3%	0.033	181	20.9% ± 1.4%	<0.001	78.1 ± 1.4%	<0.001
20.0–50.0	226	0	0.0% ± 0.0%		52	24.4% ± 3.0%		75.6 ± 3.0%	
≥50.0	302	7	2.1% ± 1.1%		94	33.2% ± 2.8%		64.7 ± 2.9%	
CNS disease									
Negative	1425	17	1.0% ± 0.3%	0.595	308	23.5% ± 1.2%	0.027	75.5 ± 1.2%	0.015
Positive	49	1	0.0% ± 0.0%		16	34.1% ± 7.0%		65.9 ± 7.0%	
Immunophenotype									
BCP‐ALL	1237	16	1.1% ± 0.3%	0.929	256	22.8% ± 1.3%	0.1	76.1 ± 1.3%	0.097
T‐ALL	198	3	1.0% ± 0.9%		49	25.6% ± 3.2%		73.5 ± 3.3%	
Genetics									
*ETV6::RUNX1*									
Positive	264	4	1.9% ± 0.9%	0.416	27	12.5% ± 2.4%	<0.001	85.6 ± 2.5%	<0.001
Negative	953	9	1.1% ± 0.4%		196	22.6% ± 1.4%		76.3 ± 1.5%	
*TCF3::PBX1*									
Positive	37	0	0.0% ± 0.0%	0.531	4	11.6 ± 5.5%	0.26	88.4 ± 5.5%	0.215
Negative	1180	13	1.3% ± 0.4%		219	20.7 ± 1.3%		78.0 ± 1.3%	
*BCR::ABL1*									
Positive	24	0	0.0% ± 0.0%	0.637	14	67.4% ± 11.3%	<0.001	32.6 ± 11.3%	<0.001
Negative	1193	13	1.3% ± 0.4%		209	19.5% ± 1.2%		79.2 ± 1.3%	
*KMT2A*‐rearrangement									
Positive	31	1	0.0% ± 0.0%	0.38	10	33.8% ± 8.8%	0.029	66.2 ± 8.8%	0.012
Negative	1186	12	1.3% ± 0.4%		213	20.0% ± 1.3%		78.6 ± 1.3%	
High‐hyperdiploidy									
Positive	292	2	1.2% ± 0.8%	0.521	44	17.5% ± 2.5%	0.062	81.3 ± 2.6%	0.048
Negative	820	10	1.3% ± 0.5%		161	21.9% ± 1.6%		76.8 ± 1.6%	

*Note*: Analyses were only performed for those parameters with available results.

Abbreviations: BCP, B‐cell precursor; CI, cumulative incidence; CNS, central nervous system; EFS, event‐free survival; Pts, patients; SMN, secondary malignant neoplasm; WBC count, white blood cell count.

**TABLE 2B:** 10‐year CI of an SMN and competing events and event‐free survival according to early response and final risk group

		**Secondary malignancies**			**Competing events**			**Event‐free survival**	
**Parameters **	**Pts**.	**Events**	**10‐year CI**	***p*‐Value**	**Events**	**10‐year CI**	***p*‐Value**	**10‐year EFS**	**p‐value**
Prednisone response									
Good	1090	11	1.2% ± 0.4%	0.456	183	18.9% ± 1.3%	<0.001	79.0 ± 1.3%	<0.001
Poor	117	2	1.9% ± 1.9%		37	33.8% ± 4.6%		64.3 ± 4.8%	
BM response on day 15									
M1	607	5	1.2% ± 0.6%	0.973	73	13.9% ± 1.6%	<0.001	84.90 ± 1.6%	<0.001
M2	316	3	1.5% ± 0.9%		72	26.2% ± 2.7%		72.4 ± 2.8%	
M3	118	1	1.1% ± 1.1%		40	39.3% ± 5.0%		59.6 ± 5.1%	
Remission status on day 33									
CR	1176	13	1.3% ± 0.4%	0.585	199	19.9% ± 1.2%	<0.001	79.7 ± 1.3%	<0.001
No CR	30	0	0.0% ± 0.0%		15	51.5% ± 9.4%		48.5 ± 9.4%	
MRD group									
Low‐risk	156	2	4.3% ± 3.5%	0.732	9	7.8% ± 2.5%	<0.001	87.8 ± 4.3%	<0.001
Intermediate‐risk	362	4	1.2% ± 0.7%		43	17.6% ± 3.0%		81.2 ± 3.0%	
High‐risk	32	0	0.0% ± 0.0%		9	31.5% ± 8.9%		68.5 ± 8.9%	
Final risk group									
Standard‐risk	514	5	0.4% ± 0.3%	0.265	102	20.8% ± 1.9%	<0.001	78.8 ± 1.9%	<0.001
Intermediate‐risk	756	11	1.5% ± 0.5%		127	19.2% ± 1.6%		79.4 ± 1.6%	
High‐risk	187	3	1.8% ± 1.3%		72	41.0% ± 3.8%		56.9 ± 3.9%	

*Note*: Analyses were only performed for those parameters with available results.

Abbreviations: BM, bone marrow; CI, cumulative incidence; CR, complete remission; EFS, event‐free survival; MRD, minimal residual disease; Pts, patients; SMN, secondary malignant neoplasm.

**TABLE 2C:** 10‐year CI of an SMN and competing events and event‐free survival according to radio‐ and chemotherapy

		**Secondary malignancies**			**Competing events**			**Event‐free survival**	
**Parameters**	**Pts**.	**Events**	**10‐year CI**	***p*‐Value**	**Events**	**10‐year CI**	***p*‐Value**	**10‐year EFS**	p‐value
Cyclophosphamide									
< 3.000 mg/m^2^	82	1	8.1% ± 7.7%	0.468	19	27.1% ± 5.4%		64.8 ± 9.0%	
≥3.000 mg/m^2^	1384	18	1.0% ± 0.3%		291	22.7% ± 1.2%	0.193	76.3 ± 1.2%	0.144
Cranial radiotherapy									
Yes	671	14	1.2% ± 0.5%	0.295	190	29.0% ± 1.8%	<0.001	69.7 ± 1.8%	<0.001
No	783	5	0.8% ± 0.3%		119	18.1% ± 1.6%		81.1 ± 1.6%	
VP‐16/VM‐26									
Yes	161	2	2.3% ± 1.7%	0.615	60	40.4% ± 4.1%		57.4 ± 4.3%	
No	1305	17	1.0% ± 0.3%		250	20.9% ± 1.2%	<0.001	78.1 ± 1.2%	<0.001

*Note*: Analyses were only performed for those parameters with available results.

Abbreviations: CI, cumulative incidence; EFS, event‐free survival; Pts, patients; SMN, secondary malignant neoplasm; VM‐26, teniposide; VP‐16, etoposide.

14/671 and 5/783 patients with and without CRT developed an SMN with a 10‐year CI of 1.2% ± 0.5% and 0.8% ± 0.3%, respectively (*p* = 0.295, Figure S5). Seven of nine patients who developed a CNS tumor had initially been treated with CRT (12 Gy: *n* = 3; 18 Gy: *n* = 4), whereas in the hematologic SMN group, only two of six patients had previously received CRT (12 Gy: *n* = 1, 24 Gy: *n* = 1). All patients with “other” SMNs had been initially treated with CRT (12 Gy: *n* = 2; 18 Gy: *n* = 2).

Regarding cytotoxic drugs, all patients who developed an SMN had previously received cyclophosphamide, which had been combined with CRT in two of six patients with hematologic, eight of nine patients with CNS and all patients with “other” SMNs, respectively. Only one patient each of the hematologic and the “other” SMN group had received VP‐16/VM‐26. The 10‐year OS rate for the 19 patients with an SMN was 55.0% ± 12.7%.

The continuously improving treatment strategies in pediatric ALL have led to a growing cohort of long‐term survivors, making it pivotal to seriously consider late effects. SMNs belong to the most devastating consequences of the childhood ALL treatment. Herein, we assessed the incidence, type, and outcome of SMNs after the primary pediatric ALL treatment with BFM‐based regimens in Austria over a period of 30 years, putting the focus on identifying risk factors.

We found a 10‐year CI of 1.1% ± 0.3% for the development of an SMN, which is comparable to previous reports [12, 13, 14, 15, 16, 17]. Nevertheless, long‐term follow‐up studies suggest that the CI of SMNs usually does not reach a plateau, thus, continued follow‐up of our patient cohort is still necessary [18, 19, 20]. Our analyses did not show statistically significant differences in the CI of an SMN with regard to initial characteristics of the primary ALL, response criteria, and therapy‐related factors. In particular, we did not find a significant relation between CNS disease, female gender, or younger age at primary ALL diagnosis and a higher CI of SMNs, as has been previously described [12, 14, 18, 20]. While our analyses could suggest an increasing incidence of SMNs in the more recent as compared to the earlier treatment era, the incidence rates were not statistically different and, possibly, capture of late events such as SMNs may have been missed in the earlier times. However, our analysis showed that patients with leukocyte counts ≥50.0 G/L at ALL diagnosis had a significantly higher CI of an SMN (2.1% ± 1.1%) than children with lower counts which is hard to interpret. Hijiya et al. also analyzed the relationship between leukocytes and risk of SMNs, but could not find a statistically significant relevance of this parameter [18].

A clear relationship between SMNs and previous irradiation therapy has been repeatedly described in the literature [14, 21, 22, 23]. Our findings are consistent with that, considering that 75% of patients who developed an SMN underwent CRT, in contrast to 45% of patients without an SMN. However, probably due to the low number of patients, the 10‐year CI of an SMN was not significantly different between patients with and without CRT (1.2% ± 0.5% vs. 0.8% ± 0.3%; *p* = 0.295). Notably, the incidence of an SMN continued to increase for the irradiated patients, whereas there was a plateau after 10 years in patients without irradiation. This might be explained by the fact that especially brain tumors develop with a longer latency compared to other SMNs, in particular myeloid neoplasms, and CRT is the strongest risk factor for secondary brain tumors. Several chemotherapeutic agents, especially alkylating agents and topoisomerase‐II inhibitors have been accused of increasing the risk for SMNs, particularly, of secondary myeloid neoplasms [24, 25, 26]. In our study, however, we could not observe any significant relations between VP‐16/VM‐26 or cyclophosphamide and a higher incidence of SMNs. This might be because BFM‐based ALL protocols since their introduction have mainly relied on VP‐16/VM‐26‐free chemotherapy regimens [9, 10, 11]. Importantly, in a recent report of the childhood cancer survivor study, it was shown that in survivors treated in recent eras without CRT and low doses of anthracyclines and alkylating agents, risk of SMNs was decreased and even not significantly different from the general population [27].

As our study included patients from as early as 1981 covering six trials, some SMNs may have been missed and detailed family histories indicating a cancer predisposition syndrome, leading to genetic germline investigations, are lacking, which has certainly resulted in the failure to elucidate an underlying cancer predisposition syndrome in either of the 19 patients. Nevertheless, the excellent cooperation between competent pediatric tertiary‐care oncologic centers in Austria enabled the nearly 100% complete registration of all children and adolescents up to 18 years of age in the ALL‐BFM trials since 1981, thus, providing well‐documented population‐based data with long‐term follow‐ups.

In conclusion, our results show a low risk of developing an SMN after BFM‐based treatment protocols for primary ALL. Although a moderate outcome, the 5‐year OS of 55.0% ± 12.7% of the SMNs, with 12 of 19 patients still alive, suggests treating these patients as aggressively as children with primary analogous malignancies. However, future strategies should aim at identifying ALL patients at risk rigorously, such as children with cancer predisposition syndromes and immunodeficiencies, in order to adapt chemotherapy (i.e., alkylating agents, anthracyclines), if justified by growing evidence to have an effect and without losing anti‐leukemic efficacy. Furthermore, consortia should aim to establish standardized surveillance programs to detect SMNs as early as possible, especially in these at‐risk populations [3]. This may help increasing OS rates, as SMNs still are a prominent non‐relapse cause of death among pediatric ALL survivors.

## CONFLICT OF INTEREST

The authors declare that there is no conflict of interest that could be perceived as prejudicing the impartiality of the research reported.

## ETHICS STATEMENT

Studies were conducted according to the Declaration of Helsinki, approved by the respective ethics committees and, since trial ALL‐BFM‐A 2000, registered at clinicaltrials.gov (NCT00430118).

## AUTHORS’ CONTRIBUTION

Fiona Poyer and Andishe Attarbaschi were involved in designing and planning the study. Fiona Poyer, Ulrike Pötschger, and Andishe Attarbaschi wrote the manuscript. Michael Dworzak, Melanie Tamesberger, Neil Jones, Reinhard Moser, Christian Urban, Georg Mann, and Andishe Attarbaschi were principal or co‐investigators in their institutions, provided study materials and recruited patients. Karin Dieckmann was in charge of cranial radiotherapy planning when indicated, and Michael Dworzak and Stefan Köhrer were in charge of minimal residual disease analysis. Christina Peters was the reference physician for allogeneic hematopoietic stem cell transplantations in patients having an indication in first remission and Oskar Haas and Karin Nebral were in charge of genetic analysis. Ulrike Pötschger performed the statistical analyses. Fiona Poyer, Georg Mann, and Andishe Attarbaschi oversaw data checking, pooling, and reporting during the study period and analyzed the data. All authors have approved the final version of the manuscript.

## Supporting information

Supporting Information

## Data Availability

All data pertinent to this work are available by contacting Andishe Attarbaschi at Andishe.attarbaschi@stanna.at
