# Supplementary material for: Second malignant neoplasms after treatment of 1487 children and adolescents with acute lymphoblastic leukemia—A population‐based analysis of the Austrian ALL‐BFM Study Group
Source: EJHaem. 2022 Jun 12;3(3):940–8. doi: 10.1002/jha2.488 (PMC9421960; doi:10.1002/jha2.488)

**Second malignant neoplasms after treatment of 1487 children and adolescents with acute lymphoblastic leukemia – A population-based analysis of the Austrian ALL-BFM Study Group**

Poyer and Attarbaschi, et al.

Supporting Information

Table of contents page

List of participating centers and investigators 3

Treatment protocols of trial ALL-BFM-A 81 4

Treatment protocols of trial ALL-A 84 5

Treatment protocols of trial ALL-BFM-A 86 6

Treatment protocols of trial ALL-BFM-A 90 7

Treatment protocols of trial ALL-BFM -A 95 8

Treatment protocols of trial ALL-BFM-A 2000 9

# Indications and dose of preventive cranial radiotherapy 11

Cumulative doses of chemotherapeutic drugs 13

Follow-up of patients included in the 6 BFM-based ALL trials in Austria 18

Characteristics of the 4 relapsed ALL patients with an SMN 19

Fine and Grey model for secondary malignancies with relapse and

death as competing events 20

Cumulative incidence Figures S1-3, S4, S5 21

# List of participating centers and investigators (ALL-BFM-A 2000)

**BFM (Berlin-Frankfurt-Münster)-Austria**

- Division of Pediatric Hematology and Oncology and Stem Cell Transplantation, Department of Pediatrics and Adolescent Medicine, Medical University of Innsbruck, Innsbruck, Austria (Univ. Prof. Dr. B. Meister)
- Department of Pediatric Hematology and Oncology, St. Anna Children’s Hospital, Medical University of Vienna, Vienna, Austria (Univ. Prof. Dr. H. Gadner, Doz. Dr. G. Mann, Doz. Dr. A. Attarbaschi)
- Department of Pediatrics and Adolescent Medicine, State Hospital Leoben, Leoben, Austria (Univ. Prof. Dr. R. Kerbl, Dr. R. Moser)
- Department of Pediatrics and Adolescent Medicine, Kepler University Hospital Linz, Linz, Austria (Univ. Prof. Dr. K. Schmitt, Dr. G. Ebetsberger-Dachs)
- Department of Pediatrics and Adolescent Medicine, University Clinics Salzburg, Salzburg, Austria (Univ. Prof. Dr. W. Sperl, Dr. N. Jones)
- Division of Pediatric Hematology and Oncology, Department of Pediatrics and Adolescent Medicine, Medical University of Graz, Graz, Austria (Univ. Prof. Dr. C. Urban, Univ. Prof. Dr. H. Lackner)
- Department of Pediatrics and Adolescent Medicine, State Hospital Klagenfurt, Klagenfurt, Austria (Univ. Prof. Dr. W. Kaulfersch)
- Department of Pediatrics and Adolescent Medicine, State Hospital Dornbirn, Dornbirn, Austria (Univ. Prof. Dr. B. Ausserer)

**Table S1: Treatment protocols of trial ALL-BFM-A 81**

| **Treatment phase / drug** | | **Dose** | **Given on Days** |
| --- | --- | --- | --- |
| **Protocol I/1** | |  |  |
|  | Prednisone p.o. | 60 mg/m² | 1-28 |
|  | Vincristine i.v. | 1.5 mg/m² | 1,8,15,22 |
|  | Daunorubicine i.v. | 30 mg/m² | 1,8,15,22 |
|  | L-Asparaginase i.v. | 5000 E/m² | 8-21 |
| **Protocol I/2** | |  |  |
|  | Cyclophosphamide i.v. | 1000 mg/m² | 29,57 |
|  | Cytarabine i.v. | 75 mg/m² | 31-34,38-41,45-48,52-55 |
|  | 6-Mercaptopurine p.o. | 60 mg/m² | 29-56 |
|  | Methotrexate i.t. | age-dependent | 31,38,45,52 |
| **Consolidation Therapy** | |  |  |
|  | 6-Mercaptopurine p.o. | 25 mg/m² | 1-56 |
|  | Methotrexate p.o. | 25 mg/m² | 1-56 |
| **Protocol III** | |  |  |
|  | Dexamethasone p.o. | 10 mg/m² | 1-14 |
|  | Vincristine i.v. | 1.5 mg/m² | 1,8 |
|  | Adriamycin i.v. | 30 mg/m² | 1,8 |
|  | L-Asparaginase i.v. | 10000 E/m² | 1,4,8,11 |
|  | Cytarabine i.v. | 75 mg/m² | 17-20,24-27 |
|  | 6-Thioguanine p.o. | 60 mg/m² | 15-28 |
|  | Methotrexate i.t. | age-dependent | 17,24 |
| **Protocol II** | |  |  |
|  | Dexamethasone p.o. | 10 mg/m² | 1-28 |
|  | Vincristine i.v. | 1.5 mg/m² | 1,8,15,22 |
|  | Adriamycin i.v. | 30 mg/m² | 1,8,15,22 |
|  | L-Asparaginase i.v. | 10000 E/m² | 1,4,8,11 |
|  | Cyclophosphamide i.v. | 1000 mg/m² | 29 |
|  | Cytarabine i.v. | 75 mg/m² | 31-34,38-41 |
|  | Methotrexate i.t. | age-dependent | 31 |
|  | 6-Thioguanine p.o. | 60 mg/m² | 29-42 |
| **Protocol IV** | |  |  |
|  | VM-26 i.v. | 165 mg/m² | 1,4,8,11 |
|  | Cytarabine i.v. | 300 mg/m² | 1,4,8,11 |
|  | Dexamethasone p.o. | 10 mg/m² | 15-42 |
|  | Vincristine i.v. | 1.5 mg/m² | 15,22,29,36 |
|  | Adriamycin i.v. | 75 mg/m² | 15,22,29,36 |
|  | L-Asparaginase i.v. | 10000 E/m² | 15,18,22,25 |
|  | Cytarabine i.v. | 60 mg/m² | 45-48,52-55 |
|  | Methotrexate i.t. | age-dependent | 45,52 |
|  | 6-Thioguanine p.o. | 60 mg/m² | 43-56 |
| **Maintenance Therapy** | |  |  |
|  | 6-Mercaptopurine p.o. | 50 mg/m² | 1x/day |
|  | Methotrexate p.o. | 20 mg/m² | 1x/week |

**Table S2: Treatment protocols of trial ALL-A 84**

| **Treatment phase / drug** | | **Dose** | **Given on Days** |
| --- | --- | --- | --- |
| **Protocol I/1** | |  |  |
|  | Prednisone p.o. | 60 mg/m² | 1-28 |
|  | Vincristine i.v. | 1.5 mg/m² | 1,8,15,22 |
|  | Daunorubicine i.v. | 30 mg/m² | 1,8,15,22 |
|  | L-Asparaginase i.v. | 5000 E/m² | 8,11,15,18,22,25 |
|  | Methotrexate i.t. | age-dependent | 1 |
| **Protocol I/2** | |  |  |
|  | Cyclophosphamide i.v. | 1000 mg/m² | 29,57 |
|  | Cytarabine i.v. | 75 mg/m² | 31-34,38-41,45-48,52-55 |
|  | 6-Mercaptopurine p.o. | 60 mg/m² | 29-56 |
|  | Methotrexate i.t. | age-dependent | SRG/MRG:31; HRG: 31,45 |
| **Consolidation Therapy SR/MR** | |  |  |
|  | 6-Mercaptopurine p.o. | 25 mg/m² | 1-56 |
|  | MD-Methotrexate i.v. | 500 mg/m² | 2,4,6,8 |
|  | Methotrexate i.t. | age-dependent | 2,4,6,8 |
| **Consolidation Therapy HR** | |  |  |
|  | Capizzi regimen: |  |  |
|  | HD-Cytarabine i.v. | 3000 mg/m² | week 11, 14 |
|  | L-Asparaginase i.m. | 10000 E/m² | week 11, 14 |
|  | Plus |  |  |
|  | 6-Mercaptopurine p.o. | 25 mg/m² | week 17-19 |
|  | MD-Methotrexate i.v. | 1000 mg/m² | week 17-19 |
|  | Methotrexate i.t. | age-dependent | week 17-19 |
| **Protocol III** | |  |  |
|  | Dexamethasone p.o. | 10 mg/m² | 1-14 |
|  | Vincristine i.v. | 1.5 mg/m² | 1,8 |
|  | Adriamycin i.v. | 30 mg/m² | 1,8 |
|  | L-Asparaginase i.v. | 10000 E/m² | 1,4,8,11 |
|  | Cytarabine i.v. | 75 mg/m² | 17-20,24-27 |
|  | 6-Thioguanine p.o. | 60 mg/m² | 15-28 |
| **Protocol II** | |  |  |
|  | Dexamethasone p.o. | 10 mg/m² | 1-28 |
|  | Vincristine i.v. | 1.5 mg/m² | 1,8,15,22 |
|  | Adriamycin i.v. | 30 mg/m² | 1,8,15,22 |
|  | L-Asparaginase i.v. | 10000 E/m² | 1,4,8,11 |
|  | Cyclophosphamide i.v. | 1000 mg/m² | 29 |
|  | Cytarabine i.v. | 75 mg/m² | 31-34,38-41 |
|  | Methotrexate i.t. | age-dependent | 31 |
|  | 6-Thioguanine p.o. | 60 mg/m² | 29-42 |
| **Protocol IV** | |  |  |
|  | VM-26 i.v. | 165 mg/m² | 1,4,8,11 |
|  | Cytarabine i.v. | 300 mg/m² | 1,4,8,11 |
|  | Dexamethasone p.o. | 10 mg/m² | 15-42 |
|  | Vincristine i.v. | 1.5 mg/m² | 15,22,29,36 |
|  | Adriamycin i.v. | 75 mg/m² | 15,22,29,36 |
|  | L-Asparaginase i.v. | 10000 E/m² | 15,18,22,25 |
|  | Cytarabine i.v. | 60 mg/m² | 45-48,52-55 |
|  | Methotrexate i.t. | age-dependent | 45,52 |
|  | 6-Thioguanine p.o. | 60 mg/m² | 43-56 |
| **Maintenance Therapy** | |  |  |
|  | 6-Mercaptopurine p.o. | 50 mg/m² | 1x/day |
|  | Methotrexate p.o. | 20 mg/m² | 1x/week |

**Table S3: Treatment protocols of trial ALL-BFM-A 86**

| **Treatment phase / drug** | | **Dose** | **Given on Days** |
| --- | --- | --- | --- |
| **Protocol I/1** | |  |  |
|  | Prednisone p.o. | 60 mg/m² | 1-28 |
|  | Vincristine i.v. | 1.5 mg/m² | 8,15,22,29 |
|  | Daunorubicine i.v. | 40 mg/m² | 8,15,22,29 |
|  | L-Asparaginase i.v. | 10000 E/m² | 19,22,25,28,31,34,37,40 |
|  | Methotrexate i.t. | age-dependent | 1 |
| **Protocol I/2** | |  |  |
|  | Cyclophosphamide i.v. | 1000 mg/m² | 43,71 |
|  | Cytarabine i.v. | 75 mg/m² | 45-48,52-55,59-62,66-69 |
|  | 6-Mercaptopurine p.o. | 60 mg/m² | 43-70 |
|  | Methotrexate i.t. | age-dependent | 45,59 |
| **Protocol M** | |  |  |
|  | 6-Mercaptopurine p.o. | 25 mg/m² | 1-57 |
|  | HD-Methotrexate i.v. | 5000 mg/m² | 8,22,36,50 |
|  | Methotrexate i.t. | age-dependent | 8,22,36,50 |
| **Protocol E** | |  |  |
|  | Prednisone p.o. | 100 mg/m² | 1-7,15-21,29-35,43-49 |
|  | HD-Methotrexate i.v. | 5000 mg/m² | 8,22,36,50 |
|  | HD-Cytarabine i.v. | 2000 mg/m² | 1,2,29,30 |
|  | Ifosfamide i.v. | 1000 mg/m² | 15,16,43,44 |
|  | Mitoxantrone p.o. | 10 mg/m² | 1,15,29,43 |
|  | Methotrexate i.t. | age-dependent | 8,22,36,50 |
| **Protocol II** | |  |  |
|  | Dexamethasone p.o. | 10 mg/m² | 1-28 |
|  | Vincristine i.v. | 1.5 mg/m² | 8,15,22,29 |
|  | Adriamycin i.v. | 30 mg/m² | 8,15,22,29 |
|  | L-Asparaginase i.v. | 10000 E/m² | 8,11,15,18 |
|  | Cyclophosphamide i.v. | 1000 mg/m² | 36 |
|  | Cytarabine i.v. | 75 mg/m² | 38-41,45-48 |
|  | Methotrexate i.t. | age-dependent | 38,45 |
|  | 6-Thioguanine p.o. | 60 mg/m² | 36-49 |
| **Maintenance Therapy** | |  |  |
|  | 6-Mercaptopurine p.o. | 50 mg/m² | 1x/day |
|  | Methotrexate p.o. | 20 mg/m² | 1x/week |

**Table S4: Treatment protocols of trial ALL-BFM-A 90**

| **Treatment phase / drug** | | **Dose** | **Given on Days** |
| --- | --- | --- | --- |
| **Protocol I/1** | |  |  |
|  | Prednisone p.o. | 60 mg/m² | 1-28 |
|  | Vincristine i.v. | 1.5 mg/m² | 8,15,22,29 |
|  | Daunorubicine i.v. | 30 mg/m² | 8,15,22,29 |
|  | L-Asparaginase i.v. | 10000 E/m² | 12,15,18,21,24,27,30,33 |
|  | Methotrexate i.t. | age-dependent | 1,15,29 |
| **Protocol I/2** | |  |  |
|  | Cyclophosphamide i.v. | 1000 mg/m² | 36,64 |
|  | Cytarabine i.v. | 75 mg/m² | 38-41,45-48,52-55,59-62 |
|  | 6-Mercaptopurine p.o. | 60 mg/m² | 36-63 |
|  | Methotrexate i.t. | age-dependent | 45,59 |
| **Protocol M** | |  |  |
|  | 6-Mercaptopurine p.o. | 25 mg/m² | 1-57 |
|  | HD-Methotrexate i.v. | 5000 mg/m² | 8,22,36,50 |
|  | Methotrexate i.t. | age-dependent | 8,22,36,50 |
| **Protocol M-A** | |  |  |
|  | 6-Mercaptopurine p.o. | 25 mg/m² | 1-57 |
|  | HD-Methotrexate i.v. | 5000 mg/m² | 8,22,36,50 |
|  | Methotrexate i.t. | age-dependent | 8,22,36,50 |
|  | L-Asparaginase i.m. | 25000 E/m² | 54 h after HD-MTX |
| **Protocol II** | |  |  |
|  | Dexamethasone p.o. | 10 mg/m² | 1-28 |
|  | Vincristine i.v. | 1.5 mg/m² | 8,15,22,29 |
|  | Adriamycin i.v. | 30 mg/m² | 8,15,22,29 |
|  | L-Asparaginase i.v. | 10000 E/m² | 8,11,15,18 |
|  | Cyclophosphamide i.v. | 1000 mg/m² | 36 |
|  | Cytarabine i.v. | 75 mg/m² | 38-41,45-48 |
|  | Methotrexate i.t. | age-dependent | 38,45 |
|  | 6-Thioguanine p.o. | 60 mg/m² | 36-49 |
| **HR-1** | |  |  |
|  | Dexamethasone p.o. | 20 mg/m² | 1-5 |
|  | 6-Mercaptopurine p.o. | 100 mg/m² | 1-5 |
|  | Vincristine i.v. | 1.5 mg/m² | 1,6 |
|  | HD-Methotrexate i.v. | 5000 mg/m² | 1 |
|  | MTX/ARA-C/PRED i.t. | age-dependent | 1 |
|  | HD-Cytarabine i.v. | 2000 mg/m² | 5 |
|  | L-Asparaginase i.m. | 25000 E/m² | 6 |
| **HR-2** | |  |  |
|  | Dexamethasone p.o. | 20 mg/m² | 1-5 |
|  | 6-Thioguanine p.o. | 100 mg/m² | 1-5 |
|  | Vindesine i.v. | 3 mg/m² | 1 |
|  | HD-Methotrexate i.v. | 5000 mg/m² | 1 |
|  | MTX/ARA-C/PRED i.t. | age-dependent | 1 |
|  | Ifosfamide i.v. | 400 mg/m² | 1-5 |
|  | Daunorubicine i.v. | 50 mg/m² | 5 |
|  | L-Asparaginase i.m. | 25000 E/m² | 6 |
| **HR-3** | |  |  |
|  | Dexamethasone p.o. | 20 mg/m² | 1-5 |
|  | HD-Cytarabine i.v. | 2000 mg/m² | 5 |
|  | VP-16 i.v. | 150 mg/m² | 3-5 |
|  | 6-Mercaptopurine p.o. | 100 mg/m² | 1-5 |
|  | L-Asparaginase i.m. | 25000 E/m² | 6 |
|  | MTX/ARA-C/PRED i.t. | age-dependent | 1 |
| **Maintenance Therapy** | |  |  |
|  | 6-Mercaptopurine p.o. | 50 mg/m² | 1x/day |
|  | Methotrexate p.o. | 20 mg/m² | 1x/week |

**Table S5: Treatment protocols of trial ALL-BFM-A 95**

| **Treatment phase / drug** | | **Dose** | **Given on Days** |
| --- | --- | --- | --- |
| **Protocol I'/I/1** | |  |  |
|  | Prednisone p.o. | 60 mg/m² | 1-28 |
|  | Vincristine i.v. | 1.5 mg/m² | 8,15,22,29 |
|  | Daunorubicine i.v. | 30 mg/m² | I´:8,15; I: 8,15,22,29 |
|  | L-Asparaginase i.v. | 5000 E/m² | 12,15,18,21,24,27,30,33 |
|  | Methotrexate i.t. | age-dependent | 1,12,33 |
| **Protocol I'/I/2** | |  |  |
|  | Cyclophosphamide i.v. | 1000 mg/m² | 36,64 |
|  | Cytarabine i.v. | 75 mg/m² | 38-41,45-48,52-55,59-62 |
|  | 6-Mercaptopurine p.o. | 60 mg/m² | 36-63 |
|  | Methotrexate i.t. | age-dependent | 45,59 |
| **Protocol M** | |  |  |
|  | 6-Mercaptopurine p.o. | 25 mg/m² | 1-57 |
|  | HD-Methotrexate i.v. | 5000 mg/m² | 8,22,36,50 |
|  | Methotrexate i.t. | age-dependent | 8,22,36,50 |
| **Protocol M-A** | |  |  |
|  | 6-Mercaptopurine p.o. | 25 mg/m² | 1-57 |
|  | HD-Methotrexate i.v. | 5000 mg/m² | 8,22,36,50 |
|  | Methotrexate i.t. | age-dependent | 8,22,36,50 |
|  | LD-Cytarabine i.v. | 200 mg/m² | 9,23,37,51 |
| **Protocol II** | |  |  |
|  | Dexamethasone p.o. | 10 mg/m² | 1-28 |
|  | Vincristine i.v. | 1.5 mg/m² | 8,15,22,29 |
|  | Doxorubicine i.v. | 30 mg/m² | 8,15,22,29 |
|  | L-Asparaginase i.v. | 10000 E/m² | 8,11,15,18 |
|  | Cyclophosphamide i.v. | 1000 mg/m² | 36 |
|  | Cytarabine i.v. | 75 mg/m² | 38-41,45-48 |
|  | Methotrexate i.t. | age-dependent | 38,45 |
|  | 6-Thioguanine p.o. | 60 mg/m² | 36-49 |
| **HR-1** | |  |  |
|  | Dexamethasone p.o. | 20 mg/m² | 1-5 |
|  | Vincristine i.v. | 1.5 mg/m² | 1,6 |
|  | HD-Methotrexate i.v. | 5000 mg/m² | 1 |
|  | MTX/ARA-C/PRED i.t. | age-dependent | 1 |
|  | Cyclophosphamide i.v. | 200 mg/m² | 2-4 |
|  | HD-Cytarabine i.v. | 2000 mg/m² | 5 |
|  | L-Asparaginase i.m. | 25000 E/m² | 6 |
| **HR-2** | |  |  |
|  | Dexamethasone p.o. | 20 mg/m² | 1-5 |
|  | Vindesine i.v. | 3 mg/m² | 1,6 |
|  | HD-Methotrexate i.v. | 5000 mg/m² | 1 |
|  | MTX/ARA-C/PRED i.t. | age-dependent | 1 |
|  | Ifosfamide i.v. | 800 mg/m² | 2-4 |
|  | Daunorubicine i.v. | 30 mg/m² | 5 |
|  | L-Asparaginase i.m. | 25000 E/m² | 6 |
| **HR-3** | |  |  |
|  | Dexamethasone p.o. | 20 mg/m² | 1-5 |
|  | HD-Cytarabine i.v. | 2000 mg/m² | 1-2 |
|  | VP-16 i.v. | 100 mg/m² | 3-5 |
|  | 6-Mercaptopurine p.o. | 100 mg/m² | 1-5 |
|  | L-Asparaginase i.m. | 25000 E/m² | 6 |
|  | MTX/ARA-C/PRED i.t. | age-dependent | 5 |
| **Maintenance Therapy** | |  |  |
|  | 6-Mercaptopurine p.o. | 50 mg/m² | 1x/day |
|  | Methotrexate p.o. | 20 mg/m² | 1x/week |

**Table S6: Treatment protocols of trial ALL-BFM-A 2000**

| **Treatment phase / drug** | | **Dose** | **Given on Days** |
| --- | --- | --- | --- |
| **Protocol I/1 PRED** | |  |  |
|  | Prednisone p.o. | 60 mg/m² | 1-28 |
|  | Vincristine i.v. | 1.5 mg/m² | 8,15,22,29 |
|  | Daunorubicine i.v. | 30 mg/m² | 8,15,22,29 |
|  | L-Asparaginase i.v. | 5000 E/m² | 12,15,18,21,24,27,30,33 |
|  | Methotrexate i.t. | age-dependent | 1,12,33 |
| **Protocol I/1 DEXA** | |  |  |
|  | Prednisone p.o. | 60 mg/m² | 1-7 |
|  | Dexamethasone p.o. | 10 mg/m² | 8-28 |
|  | Vincristine i.v. | 1.5 mg/m² | 8,15,22,29 |
|  | Daunorubicine i.v. | 30 mg/m² | 8,15,22,29 |
|  | L-Asparaginase i.v. | 5000 E/m² | 12,15,18,21,24,27,30,33 |
|  | Methotrexate i.t. | age-dependent | 1,12,33 |
| **Protocol I/2** | |  |  |
|  | Cyclophosphamide i.v. | 1000 mg/m² | 36,64 |
|  | Cytarabine i.v. | 75 mg/m² | 38-41,45-48,52-55,59-62 |
|  | 6-Mercaptopurine p.o. | 60 mg/m² | 36-63 |
|  | Methotrexate i.t. | age-dependent | 45,59 |
| **Protocol M** | |  |  |
|  | 6-Mercaptopurine p.o. | 25 mg/m² | 1-56 |
|  | HD-Methotrexate i.v. | 5000 mg/m² | 8,22,36,50 |
|  | Methotrexate i.t. | age-dependent | 8,22,36,50 |
| **Protocol II/1** | |  |  |
|  | Dexamethasone p.o. | 10 mg/m² | 1-28 |
|  | Vincristine i.v. | 1.5 mg/m² | 8,15,22,29 |
|  | Doxorubicine i.v. | 30 mg/m² | 8,15,22,29 |
|  | L-Asparaginase i.v. | 10000 E/m² | 8,11,15,18 |
| **Protocol II/2** | |  |  |
|  | Cyclophosphamide i.v. | 1000 mg/m² | 36 |
|  | Cytarabine i.v. | 75 mg/m² | 38-41,45-48 |
|  | 6-Thioguanine p.o. | 60 mg/m² | 36-49 |
|  | Methotrexate i.t. | age-dependent | 38,45 |
| **Protocol III** | |  |  |
|  | Dexamethasone p.o. | 10 mg/m² | 1-24 |
|  | Vincristine i.v. | 1.5 mg/m² | 1,8 |
|  | Doxorubicine i.v. | 30 mg/m² | 1,8 |
|  | L-Asparaginase i.v. | 10000 E/m² | 1,4,8,11 |
|  | Cyclophosphamide i.v. | 500 mg/m² | 15 |
|  | 6-Thioguanine p.o. | 60 mg/m² | 15-28 |
|  | Cytarabine i.v. | 75 mg/m² | 17-20,24-27 |
|  | Methotrexate i.t. | age-dependent | 17,24 |
| **HR-1'** | |  |  |
|  | Dexamethasone p.o. | 20 mg/m² | 1-5 |
|  | Vincristine i.v. | 1.5 mg/m² | 1,6 |
|  | HD-Methotrexate i.v. | 5000 mg/m² | 1 |
|  | MTX/ARA-C/PRED i.t. | age-dependent | 1 |
|  | Cyclophosphamide i.v. | 200 mg/m² | 2-4 |
|  | HD-Cytarabine i.v. | 2000 mg/m² | 5 |
|  | L-Asparaginase i.v. | 25000 E/m² | 6,11 |
| **HR-2'** | |  |  |
|  | Dexamethasone p.o. | 20 mg/m² | 1-5 |
|  | Vindesine i.v. | 3 mg/m² | 1,6 |
|  | HD-Methotrexate i.v. | 5000 mg/m² | 1 |
|  | MTX/ARA-C/PRED i.t. | age-dependent | 1 |
|  | Ifosfamide i.v. | 800 mg/m² | 2-4 |
|  | Daunorubicine i.v. | 30 mg/m² | 5 |
|  | L-Asparaginase i.v. | 25000 E/m² | 6,11 |
| **HR-3'** | |  |  |
|  | Dexamethasone p.o. | 20 mg/m² | 1-5 |
|  | HD-Cytarabine i.v. | 2000 mg/m² | 1-2 |
|  | VP-16 i.v. | 100 mg/m² | 3-5 |
|  | MTX/ARA-C/PRED i.t. | age-dependent | 5 |
|  | L-Asparaginase i.v. | 25000 E/m² | 6,11 |
| **Maintenance Therapy** | |  |  |
|  | 6-Mercaptopurine p.o. | 50 mg/m² | 1x/day |
|  | Methotrexate p.o. | 20 mg/m² | 1x/week |

Table S1, S2, S3, S4, S5, and S6: p.o. indicates orally; i.v., intravenous; i.t., intrathecal.

**Indications and dose of preventive cranial radiotherapy**

**Table S7: Dose of cranial radiotherapy in trials ALL-BFM-A 81 and ALL-A 84**

|  | < 1 year | ≥ 1 and < 2 years | ≥ 2 years |
| --- | --- | --- | --- |
| SRG/MRG | 12 Gy | 15 Gy | 18 Gy |
| HRG | 15 Gy | 20 Gy | 24 Gy |

**Abbreviations**: SRG: standard-risk group; MRG: intermediate-risk group; HRG: high-risk group

**Table S8: Dose of cranial radiotherapy in trial ALL-BFM-A 86**

|  | < 1 year | ≥ 1 and < 2 years | ≥ 2 years |
| --- | --- | --- | --- |
| SRG | 0 Gy | 0 Gy | 0 Gy |
| RG (0.8-1.2) | 0 Gy | 12 Gy | 12 Gy |
| RG (≥0.8) | 0 Gy | 12 Gy | 18 Gy |
| EG | 0 Gy | 12 Gy | 18 Gy |

**Abbreviations**: SRG: standard-risk group; RG: risk group; EG: experimental group

**Table S9: Dose of cranial radiotherapy in trial ALL-BFM-A 90**

|  | < 1 year | ≥ 1 year |
| --- | --- | --- |
| SRG | 0 Gy | 0 Gy |
| MRG | 0 Gy | 12 Gy |
| HRG | 0 Gy | 12 Gy |

**Abbreviations**: SRG: standard-risk group; MRG: intermediate risk group; HRG: high-risk group

**Table S10: Dose of cranial radiotherapy in trial ALL-BFM-A 95**

|  | < 1 year | ≥ 1 year |
| --- | --- | --- |
| SRG | 0 Gy | 0 Gy |
| MRG - T-ALL | 0 Gy | 0 Gy |
| MRG + T-ALL | 0 Gy | 12 Gy |
| HRG | 0 Gy | 12 Gy |

**Abbreviations**: SRG: standard-risk group; MRG: intermediate-risk group; HRG: high-risk group

**Table S11: Dose of cranial radiotherapy in trial ALL-BFM-A 2000**

|  | < 1 year | ≥ 1 and <2 years | ≥ 2 years |
| --- | --- | --- | --- |
| T/non-HR (WBC <100 000/µl) | 0 Gy | 0 Gy | 12 Gy |
| T/non-HR (WBC ≥100 000/µl) | 0 Gy | 0 Gy | 12 Gy |
| pB/non-HR | 0 Gy | 0 Gy | 0 Gy |
| HR | 0 Gy | 12 Gy | 12 Gy |

**Abbreviations**: T: T-cell ALL; non-HR: non-high-risk; WBC: white blood cells; pB: precursor B-cell ALL; HR: high-risk;

**Cumulative doses of chemotherapeutic drugs**

**Table S12: Cumulative doses of drugs used in trial ALL-BFM-A 81**

|  | **SRG** | **MRG** | **HRG** |
| --- | --- | --- | --- |
| **DNR mg/m²** | 120 (4) | 120 (4) | 120 (4) |
| **DOX mg/m²** | 60 (2) | 120 (4) | 300 (4) |
| **CPM mg/m²** | 2 000 (2) | 3 000 (3) | 2 000 (2) |
| **IFO mg/m²** | - | - | - |
| **VP-16 mg/m²** | - | - | - |
| **VM-26 mg/m²** | - | - | 660 (4) |
| **MTX* ith. mg/m²** | 36/48/60/72 (6) | 30/40/50/60 (5) | 36/48/60/72 (6) |
| **MTX iv. mg/m²** | - | - | - |

**Abbreviations**: SRG: standard-risk group; MRG: intermediate-risk group; HRG: high-risk group

DNR: daunorubicine; DOX: doxorubicine; CPM: cyclophosphamide; IFO: ifosfamide; VP-16: etoposide;

VM-26: teniposide; MTX: methotrexate; ith: intrathecal; iv: intravenous

In parentheses: number of dosages by which cumulative dose was administered

*MTX-dose: <1 year 6 mg/ ≥1 year and <2 years 8 mg/ ≥2 years and <3 years 10 mg/ ≥3 years 12 mg

**Table S13: Cumulative doses of drugs used in trial ALL-A 84**

|  | **SRG** | **MRG** | **HRG** |
| --- | --- | --- | --- |
| **DNR mg/m²** | 120 (4) | 120 (4) | 120 (4) |
| **DOX mg/m²** | 60 (2) | 120 (4) | 300 (4) |
| **CPM mg/m²** | 2 000 (2) | 3 000 (3) | 2 000 (2) |
| **IFO mg/m²** | - | - | - |
| **VP-16 mg/m²** | - | - | - |
| **VM-26 mg/m²** | - | - | 660 (4) |
| **MTX* ith. mg/m²** | 36/48/60/72 (6) | 42/56/70/84 (7) | 48/64/80/96 (8) |
| **MTX iv. mg/m²** | 2 000 (4) | 2 000 (4) | 3 000 (3) |

**Abbreviations**: SRG: standard-risk group; MRG: intermediate-risk group; HRG: high-risk group

DNR: daunorubicine; DOX: doxorubicine; CPM: cyclophosphamide; IFO: ifosfamide; VP-16: etoposide;

VM-26: teniposide; MTX: methotrexate; ith: intrathecal; iv: intravenous

In parentheses: number of dosages by which cumulative dose was administered

*MTX-dose: <1 year 6 mg/ ≥1 year and <2 years 8 mg/ ≥2 years and <3 years 10 mg/ ≥3 years 12 mg

**Table S14: Cumulative doses of drugs used in trial ALL-BFM-A 86**

|  | **SRG (RF <0.8)** | **RG (RF ≥0.8)** | **EG** |
| --- | --- | --- | --- |
| **DNR mg/m²** | 160 (4) | 160 (4) | 160 (4) |
| **DOX mg/m²** | 120 (4) | 120 (4) | 120 (4) |
| **CPM mg/m²** | 2 000 (2) | 3 000 (3) | 3 000 (3) |
| **IFO mg/m²** | - | - | 4 000 (4) |
| **VP-16 mg/m²** | - | - | - |
| **VM-26 mg/m²** | - | - | - |
| **MTX* ith. mg/m²** | 54/72/90/108 (9) | 54/72/90/108 (9) | 54/72/90/108 (9) |
| **MTX iv. mg/m²** | 20 000 (4) | 20 000 (4) | 20 000 (4) |

**Abbreviations**: SRG: standard-risk group; RG: risk group; EG: experimental group; RF: risk factor

DNR: daunorubicine; DOX: doxorubicine; CPM: cyclophosphamide; IFO: ifosfamide; VP-16: etoposide;

VM-26: teniposide; MTX: methotrexate; ith: intrathecal; iv: intravenous

In parentheses: number of dosages by which cumulative dose was administered

*MTX-dose: <1 year 6 mg/ ≥1 year and <2 years 8 mg/ ≥2 years and <3 years 10 mg/ ≥3 years 12 mg

**Table S15: Cumulative doses of drugs used in trial ALL-BFM-A 90**

|  | **SRG** | **MRG** | **HRG** |
| --- | --- | --- | --- |
| **DNR mg/m²** | 120 (4) | 120 (4) | 270 (7) |
| **DOX mg/m²** | 120 (4) | 120 (4) | - |
| **CPM mg/m²** | 3 000 (3) | 3 000 (3) | - |
| **IFO mg/m²** | - | - | 6 000 (15) |
| **VP-16 mg/m²** | - | - | 1 350 (9) |
| **VM-26 mg/m²** | - | - | - |
| **MTX* ith. mg/m²** | 66/88/110/132 (11) | 66/88/110/132 (11) | 72/96/120/144 (12) |
| **MTX iv. mg/m²** | 20 000 (4) | 20 000 (4) | 30 000 (6) |

**Abbreviations**: SRG: standard-risk group; MRG: intermediate-risk group; HRG: high-risk group

DNR: daunorubicine; DOX: doxorubicine; CPM: cyclophosphamide; IFO: ifosfamide; VP-16: etoposide;

VM-26: teniposide; MTX: methotrexate; ith: intrathecal; iv: intravenous

In parentheses: number of dosages by which cumulative dose was administered

*MTX-dose: <1 year 6 mg/ ≥1 year and <2 years 8 mg/ ≥2 years and <3 years 10 mg/ ≥3 years 12 mg

**Table S16: Cumulative doses of drugs used in trial ALL-BFM-A 95**

|  | **SRG** | **MRG** | **HRG** |
| --- | --- | --- | --- |
| **DNR mg/m²** | 60 (2) | 120 (4) | 180 (6) |
| **DOX mg/m²** | 120 (4) | 120 (4) | 120 (4) |
| **CPM mg/m²** | 3 000 (3) | 3 000 (3) | 4 200 (9) |
| **IFO mg/m²** | - | - | 4 800 (6) |
| **VP-16 mg/m²** | - | - | 600 (6) |
| **VM-26 mg/m²** | - | - | - |
| **MTX* ith. mg/m²** | 66/88/110/132 (11) | 66/88/110/132 (11) | 78/104/130/156 (13) |
| **MTX iv. mg/m²** | 20 000 (4) | 20 000 (4) | 20 000 (4) |

**Abbreviations**: SRG: standard-risk group; MRG: intermediate-risk group; HRG: high-risk group

DNR: daunorubicine; DOX: doxorubicine; CPM: cyclophosphamide; IFO: ifosfamide; VP-16: etoposide;

VM-26: teniposide; MTX: methotrexate; ith: intrathecal; iv: intravenous

In parentheses: number of dosages by which cumulative dose was administered

*MTX-dose: <1 year 6 mg/ ≥1 year and <2 years 8 mg/ ≥2 years and <3 years 10 mg/ ≥3 years 12 mg

**Table S17: Cumulative doses of drugs used in trial ALL-BFM-A 2000**

|  | **T/non-HR** | **pB/non-HR** | **HR** |
| --- | --- | --- | --- |
| **DNR mg/m²** | 120 (4) | 120 (4) | 180 (6) |
| **DOX mg/m²** | 120 (4) | 120 (4) | 120 (4) |
| **CPM mg/m²** | 3 000 (3) | 3 000 (3) | 4 200 (9) |
| **IFO mg/m²** | - | - | 4 800 (6) |
| **VP-16 mg/m²** | - | - | 600 (6) |
| **VM-26 mg/m²** | - | - | - |
| **MTX* ith. mg/m²** | 66/88/110/132 (11) | 66/88/110/132 (11) | 78/104/130/156 (13) |
| **MTX iv. mg/m²** | 20 000 (4) | 20 000 (4) | 20 000 (4) |

**Abbreviations**: T/non-HR: T-cell ALL, non-high-risk; pB/ non-HR: pre-B-cell ALL, non-high-risk; HR: high-risk group; DNR: daunorubicine; DOX: doxorubicine; CPM: cyclophosphamide; IFO: ifosfamide; VP-16: etoposide; VM-26: teniposide; MTX: methotrexate; ith: intrathecal; iv: intravenous

In parentheses: number of dosages by which cumulative dose was administered

*MTX-dose: <1 year 6 mg/ ≥1 year and <2 years 8 mg/ ≥2 years and <3 years 10 mg/ ≥3 years 12 mg

**Table S18: Follow-up of patients included in the 5 BFM-based ALL trials in Austria**

| **Trial** | **No. of patients enrolled** | **No. of SMNs** | **Median follow-up (years)** |
| --- | --- | --- | --- |
| ALL-BFM-A 81 | 141 | 1 (0.7%) | 12.7  (range 0.2-27.6) |
| ALL-A 84 | 127 | 7 (5.5%)* | 15.0  (range 0.1-24.1) |
| ALL-BFM-A 86 | 142 | 3 (2.1%)* | 8.1  (range 3.3-21.9) |
| ALL-BFM-A 90 | 256 | 3 (1.6%) | 7.8  (range 0.5-18.4) |
| ALL-BFM-A 95 | 230 | 2 (0.9%)* | 6.4  (range 1.5-13.7) |
| ALL-BFM-A 2000 | 591 | 7 (1.2%) | 5.3  (range 0.3-12.2) |
| Total | 1487 | 23 (1.5%) | 9.5 |

**Abbreviations**: No.: number; SMN: secondary malignant neoplasm

*: 2 patients from ALL-A 84 and one patient each from ALL-BFM-A 86 and 95 had a SMN after treatment for ALL relapse.

**Table S19: Characteristics of the 4 relapsed ALL patients with a secondary malignant neoplasm**

| **Pat. Number** | **Study** | **Age at ALL (years)** | **Gender** | **Phenotype of ALL** | **Age at SMN (years)** | **Time to SMN (years)** | **Cranial radiotherapy** | **Type of SMN** | **Therapy of SMN*** | **Outcome of SMN** | **Survival time from SMN (months)** |
| --- | --- | --- | --- | --- | --- | --- | --- | --- | --- | --- | --- |
| 1 | ALL-A 84 | 13.9 | m | C-ALL | 30.1 | 16.1 | no | parotid gland tumour | 2 | alive | 80 |
| 2 | ALL-A 84 | 7.2 | m | C-ALL | 13.1 | 5.9 | 18 Gy | MDS - RAEB | 1 | dead (progression of SMN) | 4 |
| 3 | ALL-BFM-A 86 | 8.7 | m | C-ALL | 25.4 | 16.9 | 18 Gy | carcinoma of the tongue | 1,3 | dead (progression of SMN) | 19 |
| 4 | ALL-BFM-A 95 | 3.6 | f | C-ALL | 13.8 | 10.4 | 12 Gy | PNET, brain | 1,2,3 | dead (progression of SMN) | 32 |

*Therapy of SMN: 1=chemotherapy, 2=operation, 3=radiation

**Abbreviations**: Pat.: patient; SMN: secondary malignant neoplasm; m: male; f: female; C-ALL: common ALL; MDS: myelodysplastic syndrome; RAEB: refractory anaemia with excess blasts; PNET: primitive neuroectodermal tumour

**Table S20:** **Fine and Grey model for secondary malignancies with relapse and death as competing events**

|  |  | **P-value** | **Hazard ratio** | **95% Hazard Ratio Confidence Limits** | |
| --- | --- | --- | --- | --- | --- |
| **Trial** | **vs. ALL-BFM-A 2000** | NS |  |  |  |
|  | **ALL-BFM-A 81** | 0.108 | 0.13 | 0.01 | 1.56 |
|  | **ALL-A 84** | 0.492 | 0.66 | 0.21 | 2.13 |
|  | **ALL-BFM-A 86** | 0.418 | 0.56 | 0.14 | 2.26 |
|  | **ALL-BFM-A 90** | 0.099 | 0.32 | 0.08 | 1.24 |
|  | **ALL-BFM-A 95** | 0.151 | 0.20 | 0.02 | 1.78 |
| **CRT** |  | 0.105 | 2.14 | 0.85 | 5.35 |
| **Age (years)** | **≥10 vs. < 10** | 0.681 | 1.23 | 0.46 | 3.24 |
| **WBC count (G/L)** | **≥50.0 vs. < 50.0** | 0.130 | 2.03 | 0.81 | 5.07 |

**Abbreviations:** CRT, cranial radiotherapy; WBC, white blood cells; NS, not significant

**Figure S1-3: Cumulative incidences (CI) of (1) secondary malignancies with relapse and death as competing events *vs.* (2) relapse as a first event, and (3) death as a first event**


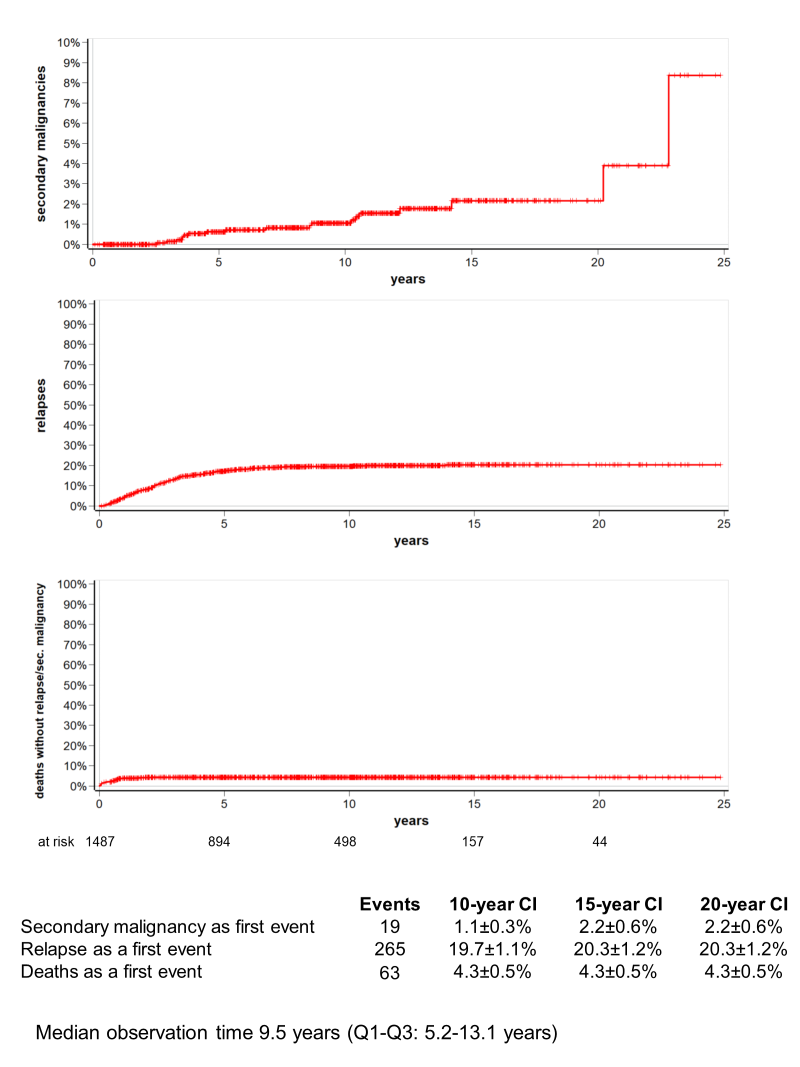


**Figure S4: Cumulative incidences of secondary malignancies with (A) relapse and death as competing events *vs.* (B) deaths only as competing events**


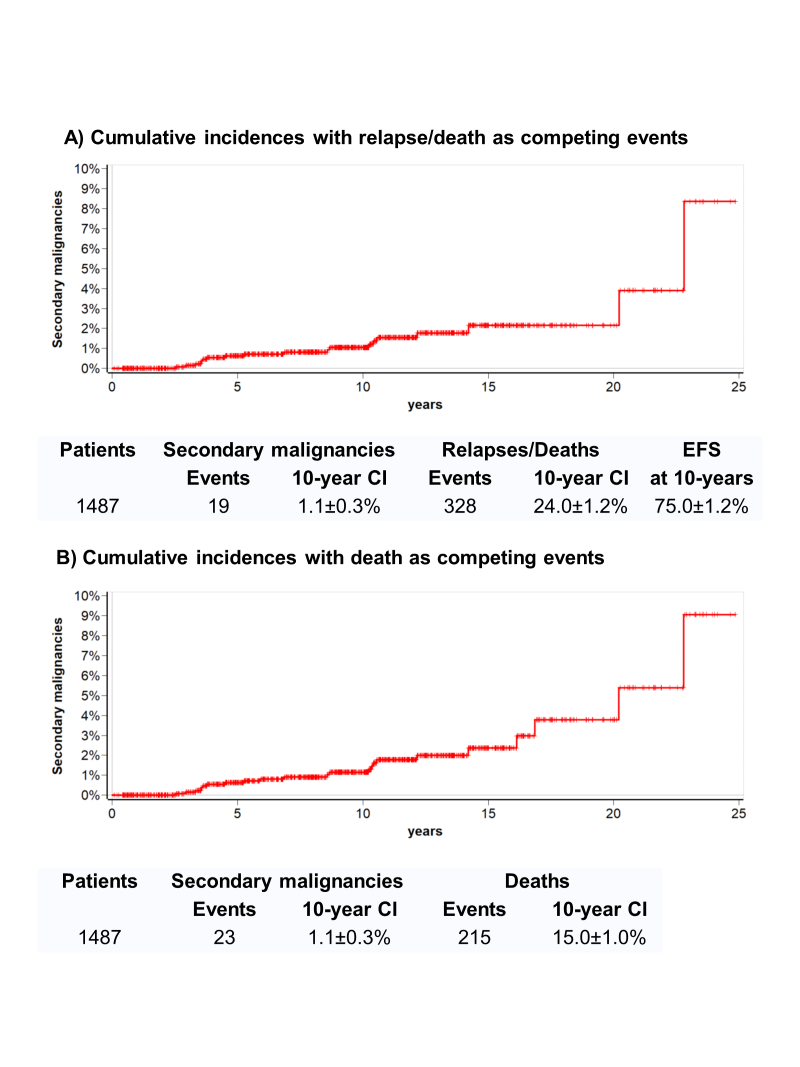


**Figure S5: Cumulative incidences of secondary malignancies with relapse and death as competing events according to the usage of cranial radiotherapy (CRT)**


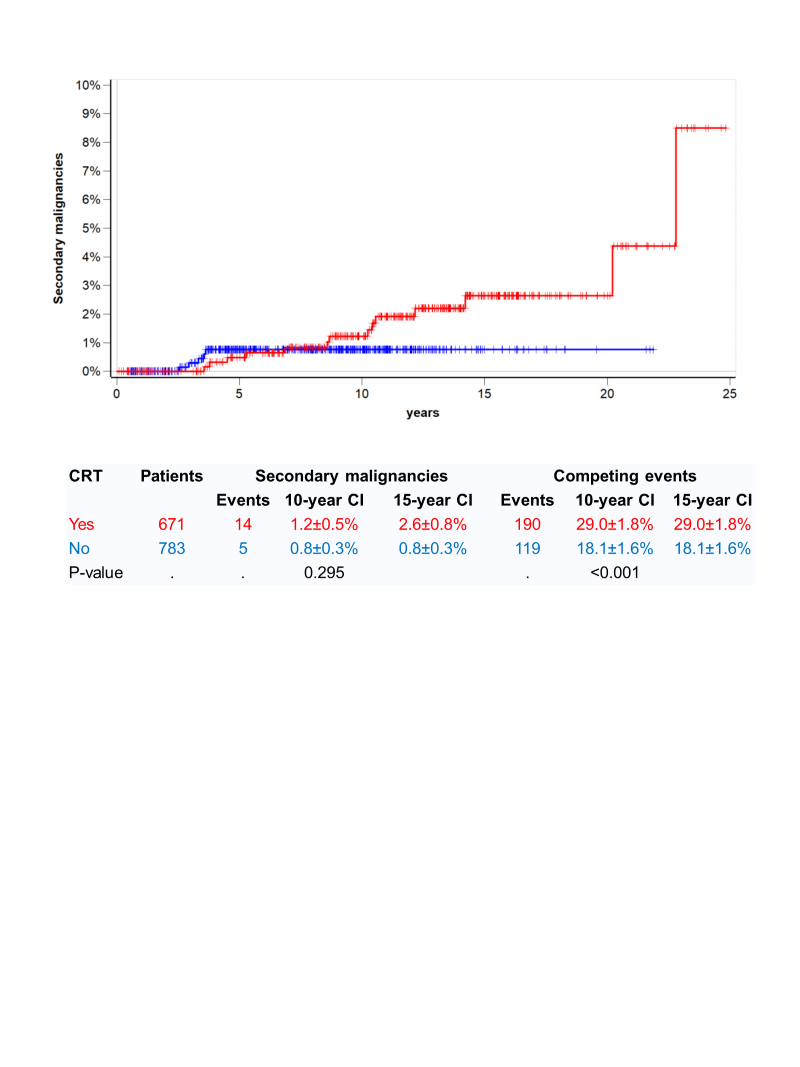

Supplement: Supplementary file 1 — Supporting Information [file JHA2-3-940-s001.docx]
